# TMS-EEG Biomarkers of Amnestic Mild Cognitive Impairment Due to Alzheimer’s Disease: A Proof-of-Concept Six Years Prospective Study

**DOI:** 10.3389/fnagi.2021.737281

**Published:** 2021-11-22

**Authors:** Florinda Ferreri, Andrea Guerra, Luca Vollero, David Ponzo, Sara Määtta, Mervi Könönen, Fabrizio Vecchio, Patrizio Pasqualetti, Francesca Miraglia, Ilaria Simonelli, Maurizio Corbetta, Paolo Maria Rossini

**Affiliations:** ^1^Unit of Neurology, Unit of Clinical Neurophysiology and Study Center of Neurodegeneration (CESNE), Department of Neuroscience, University of Padua, Padua, Italy; ^2^Department of Clinical Neurophysiology, Kuopio University Hospital, University of Eastern Finland, Kuopio, Finland; ^3^IRCCS Neuromed, Pozzilli, Italy; ^4^Department of Computer Science and Computer Engineering, Campus Bio-Medico University of Rome, Rome, Italy; ^5^Department of Clinical Radiology, Kuopio University Hospital, Kuopio, Finland; ^6^Brain Connectivity Laboratory, Department of Neuroscience and Neurorehabilitation, IRCCS San Raffaele Roma, Rome, Italy; ^7^eCampus University, Novedrate, Como, Italy; ^8^Servizio di Statistica Medica ed Information Technology, Associazione Fatebenefratelli per la Ricerca (AFaR), Rome, Italy; ^9^Department of Neuroscience, Neurology, Radiology and Biomedical Engineering, Washington University in St. Louis, St. Louis, MO, United States; ^10^Padua Neuroscience Center, University of Padua, Padua, Italy

**Keywords:** mild cognitive impairment (MCI), Alzheimer’s disease (AD), electroencephalography (EEG), navigated transcranial magnetic stimulation (nTMS), TMS-EEG coregistration

## Abstract

**Background:** Early and affordable identification of subjects with amnestic mild cognitive impairment (aMCI) who will convert to Alzheimer’s disease (AD) is a major scientific challenge.

**Objective:** To investigate the neurophysiological hallmarks of sensorimotor cortex function in aMCI under the hypothesis that some may represent the plastic rearrangements induced by neurodegeneration, hence predictors of future conversion to AD. We sought to determine (1) whether the sensorimotor network shows peculiar alterations in patients with aMCI and (2) if sensorimotor network alterations predict long-term disease progression at the individual level.

**Methods:** We studied several transcranial magnetic stimulation (TMS)-electroencephalogram (EEG) parameters of the sensorimotor cortex in a group of patients with aMCI and followed them for 6 years. We then identified aMCI who clinically converted to AD [prodromal to AD-MCI (pAD-MCI)] and those who remained cognitively stable [non-prodromal to AD-MCI (npAD-MCI)].

**Results:** Patients with aMCI showed reduced motor cortex (M1) excitability and disrupted EEG synchronization [decreased intertrial coherence (ITC)] in alpha, beta and gamma frequency bands compared to the control subjects. The degree of alteration in M1 excitability and alpha ITC was comparable between pAD-MCI and npAD-MCI. Importantly, beta and gamma ITC impairment in the stimulated M1 was greater in pAD-MCI than npAD-MCI. Furthermore, an additional parameter related to the waveform shape of scalp signals, reflecting time-specific alterations in global TMS-induced activity [stability of the dipolar activity (sDA)], discriminated npAD-MCI from MCI who will convert to AD.

**Discussion:** The above mentioned specific cortical changes, reflecting deficit of synchronization within the cortico-basal ganglia-thalamo-cortical loop in aMCI, may reflect the pathological processes underlying AD. These changes could be tested in larger cohorts as neurophysiological biomarkers of AD.

## Introduction

The term mild cognitive impairment (MCI) describes an intermediate stage in the trajectory from normal cognition to dementia (Petersen et al., 2018). A clinical presentation with memory impairment is defined as amnestic MCI (aMCI), a condition considered as a precursor of Alzheimer’s disease (AD) given the high rate of progression to dementia (Prestia et al., 2013). The ability to separate at an early-stage aMCI who will convert to AD from those who will not is an emerging scientific priority, as evidence shows that early diagnosis significantly reduces the health and social burden of dementia management (Rossini et al., 2019). To date, the diagnosis of prodromal-to-AD MCI can be reached with high sensitivity and specificity by combining different tests (e.g., hippocampal volumetric MRI, PET integrated with beta-amyloid and tau radioligands, cerebrospinal fluid beta, and tau metabolites dosage). However, due to their high costs, limited availability, and/or invasiveness, these tests cannot be used as screening tools (Rossini et al., 2019).

Over the years, several abnormalities have been detected in pathological brain aging by using electroencephalography (EEG) and transcranial magnetic stimulation (TMS) techniques. Both EEG and TMS may be suitable screening methods as they are widely available, non-invasive, and low cost (Rossini et al., 2013). This body of work has shown that generalized slowing of the brain rhythms, reduced network complexity in EEG organization (Babiloni et al., 2016), increased cortical excitability (Ferreri et al., 2003, 2011b), altered intracortical connectivity, and disrupted plasticity in TMS-perturbed networks (Guerra et al., 2015; Benussi et al., 2018; Bologna et al., 2020; Di Lorenzo et al., 2020; Colella et al., 2021) are the neurophysiological hallmarks of AD progression (Rossini et al., 2020). However, these abnormalities have been usually significant only at the group level and generally the patients were followed-up for short time after the neurophysiological assessment (de Haan et al., 2012).

We recently used TMS-EEG co-registration (Ilmoniemi et al., 1997) to describe, for the first time, stimulus-evoked changes of motor cortex (M1) function in mild patients with AD and found increased excitability and altered sensorimotor connectivity, despite no motor symptoms were present (Ferreri et al., 2016). We interpreted these findings as evidence of a plastic cortical reorganization, which may include the recruitment of additional or reverberant local neural circuits and their integration in the distributed network subtending sensorimotor functions through the multiple somatotopic maps (Sanes and Donoghue, 2000). These mechanisms may allow the preservation of sensorimotor programming and execution, since the preclinical dementia stage and over a long period of time in spite of disease progression (Ferreri et al., 2003).

In this study, we investigated the TMS-EEG correlates of aMCI in sensorimotor cortex to verify whether they predict future conversion to AD under the assumption that they represent plastic subclinical adjustments induced by neurodegeneration (Ferreri and Rossini, 2013). We studied a group of aMCI patients and, then, we followed them for 6 years. At the end of the observation time, we identified the aMCI who clinically converted to AD [prodromal to AD-MCI (pAD-MCI)] and those who remained cognitively stable [non-prodromal to AD-MCI (npAD-MCI)]. We first compared the excitability and effective connectivity of the somatosensory network in the whole aMCI group to healthy controls; then, we analyzed the possible differences in neurophysiological properties between pAD-MCI and npAD-MCI. We sought to determine: (1) whether the sensorimotor network showed peculiar alterations in the aMCI group and (2) if sensorimotor network alterations predicted long-term disease progression at the individual level.

## Materials and Methods

### Subjects

A total of 17 patients with MCI were recruited based on the diagnostic criteria for aMCI (Albert et al., 2011). The control group (CO) consisted of 15 age- and gender-matched healthy subjects. All the participants were right-handed and none showed movement abnormalities or had contraindications to the use of TMS (Rossini et al., 2015). The Research Ethics Committee approved the study protocol and the study was carried out in accordance with the latest version of the Helsinki Declaration. A total of 13 patients with aMCI completed a clinical-neuropsychological 6-years follow-up. Then, the patients whose diagnosis satisfied the clinical criteria for AD (McKhann et al., 2011) were classified as pAD-MCI, while the subjects who did not convert to AD were classified as npAD-MCI. Four patients with aMCI were lost at follow-up. All the 17 patients with aMCI underwent the TMS-EEG recording at the beginning of the follow-up period. Demographic, clinical, and neurophysiological characteristics of the participants in the study are shown in Table 1.

**TABLE 1:** Demographic, clinical, and neurophysiological characteristics of participants.

	**Controls (*n* = 15)**	**aMCI** **(*n* = 17)**	**pAD-MCI** **(*n* = 7)**	**npAD-MCI** **(*n* = 6)**	**aMCI vs. Controls**	**pAD-MCI vs. npAD-MCI**
Age, *y* (*mean* ± *SD*)	67.5 ± 7.0	70.9 ± 5.6	70.9 ± 5.3	69.5 ± 6.1	*p* = 0.12	*p* = 0.67
Gender (*F*, *M*)	7, 8	7, 10	2, 5	4, 2	*p* = 0.99	*p* = 0.28
Education, *y* (*mean* ± *SD*)	9.0 ± 4.2	7.6 ± 3.7	8.0 ± 4.7	7.2 ± 3.9	*p* = 0.31	*p* = 0.94
MMSE baseline (*mean* ± *SD*)	29.7 ± 0.3	26.7 ± 1.7	26.6 ± 1.5	26.5 ± 2.0	*p* < 0.01	*p* = 0.94
RAVLT immediate recall (*mean* ± *SD*)	–	26.8 ± 4.8	25.3 ± 3.4	28.8 ± 6.7	–	*p* = 0.43
RAVLT delayed recall (*mean* ± *SD*)	–	3.5 ± 2.0	2.7 ± 1.9	4.3 ± 2.1	–	*p* = 0.19
Neuropsychological profile	–	7 aMCI-SD; 10 aMCI-MD	1 aMCI-SD; 6 aMCI-MD	3 aMCI-SD; 3 aMCI-MD	–	–
Observation time, *m* (*median*, *range*)	–	–	52.0, 48−82	69.5, 51−80	–	–
Time-to-conversion, *m* (*median*, *range*)	–	–	24.0, 6−44	–	–	–
Delta MMSE end observation time-baseline (*mean* ± *SD*)	–	–	−6.8 ± 1.9	−1.7 ± 0.8	–	*p* < 0.01
RMT, % (*mean* ± *SD*)	57.2 ± 5.3	58.6 ± 8.6	56.9 ± 9.2	60.8 ± 6.4	*p* = 0.87	*p* = 0.39
MEP amplitude, μ*V* (*mean* ± *SD*)	579 ± 328	726 ± 548	677 ± 268	752 ± 426	*p* = 0.11	*p* = 0.51

*aMCI, patients with amnestic Mild Cognitive Impairment; pAD-MCI, aMCI who clinically converted to AD; npAD-MCI, aMCI who remained cognitively stable; MMSE, Mini Mental State Examination score; RAVLT, Rey’s Auditory Verbal Learning Test score; aMCI-SD, amnestic MCI single domain, i.e., deficit on at least 1 of the memory tests with no deficit in other domains; aMCI-MD, amnestic MCI multiple domains, i.e., at least 1 deficit in memory plus at least 1 additional deficit in another domain; RMT, resting motor threshold; MEP, motor evoked potential; SD, standard deviation; F, female; M, male; y, years; m, months.*

### Electroencephalography Recordings

A TMS-compatible EEG equipment (BrainAmp 32MRplus, BrainProducts GmbH, Munich, Germany) was used. EEG signals were acquired from 32 channels (10-10 International System), bandpass filtered at 0.1–1,000 Hz, and digitized at a sampling rate of 5 kHz (Ferreri et al., 2011a, 2016, 2017a,2017b). The ground was positioned in Oz and the linked mastoid served as reference. Skin/electrode impedance was <5 kOhms. Eye movements were detected by electro-oculogram (EOG). To mask the coil-generated clicks, a white noise was continuously delivered through earphones (always below 90 dB) (Massimini et al., 2005). A foam layer was placed between the coil and the EEG cap. To ensure wakefulness, subjects were required to fixate a target over the wall.

### Transcranial Magnetic Stimulation

Single-pulse TMS (monophasic stimuli; Magstim 200^2^, Magstim Company Ltd. Spring Gardens, Whitland, United Kingdom) was performed using a figure-of-eight coil. Each subject underwent 100 navigated TMS trials (intertrial interval 6–8 s) at 120% of the resting motor threshold (RMT) intensity over the right first dorsal interosseous (FDI) muscle hotspot. The hotspot was defined (Rossini et al., 2015) as the point from which stimuli triggered motor evoked potentials (MEPs) of maximal amplitude and minimal latency in the target muscle. The coordinates of the head, EEG electrodes, and coil were transferred to the same coordinate system with MRI scans through a neuronavigation system (SofTaxic Optic System, EMS SRL, Bologna, Italy). MEPs were recorded by using surface electrodes and measured offline.

### Data Analysis and Statistics

Electroencephalography signals were segmented in the time windows of ±1 s around the stimulus and preprocessed according to the previous studies (Ferreri et al., 2011a, 2012, 2016; Kaarre et al., 2018; Ferrarelli et al., 2019; Määttä et al., 2019). The global mean field power (GMFP) was calculated for each group (CO and aMCI, npAD-MCI, and pAD-MCI) and, for a topographical assessment, we integrated the EEG signals in a map by using the time points demonstrating a different GMFP activity between the groups (Delorme and Makeig, 2004). To evaluate the event-related changes in the frequency domain, the event-related spectral perturbation (ERSP) and intertrial coherence (ITC) were calculated (Delorme and Makeig, 2004). We extracted the average ERSP and ITC for alpha (8–13 Hz), beta (13.5–30 Hz), and gamma (30.5–80 Hz, excluding 45–55 Hz band due to notch filtering application) frequency bands. To possibly detect the simple features able in differentiating pAD-MCI from npAD-MCI both at the group and at the individual level, we extracted three additional metrics from the GMFP of each aMCI patient: (i) the number of significant local maxima (# of peaks), calculated following the methodology used in Massimini et al., 2005; (ii) the average GMFP level [average dipolar activity (aDA)]; and (iii) the SD of the GMFP [stability of the dipolar activity (sDA)]. We defined these metrics “aDA” and “sDA” since a GMFP sample (i.e., the absolute value of the EEG signals across all the electrodes in a specific time point) can be interpreted as the instantaneous dipolar activity on the scalp. High GMFP values result from the group of electrodes with highly positive (positive pole) and/or negative values (negative pole), while low GMFP values reflect the weak poles (electrode values ≈ 0). Thus, aDA indicates the average power of the dipolar activity, while sDA measures the variations of the dipolar activity power over time.

Statistical analyses were performed as follows. Gender differences between the groups were evaluated by the Fisher’s exact test. The Mann–Whitney *U* test was used to compare age, education, and the Mini-Mental State Examination (MMSE) scores. Differences in RMT and MEP amplitude were assessed with an unpaired *t*-test. The GMFPs of the different groups were checked for normality (the Kolmogorov–Smirnov test) and contrasted by using sample-by-sample independent *t*-test. Time domain [TMS-evoked potentials (TEPs)] and frequency domain (ERSP and ITC) features were evaluated by using the repeated measures (rm) ANOVAs. To compare TEPs between the aMCI and controls, we used the between-group factor “group” (2 levels: CO and aMCI) and the within-group factor “channel” (32 levels: Fp1, Fp2, F3, F4, C3, C4, P3, P4, O1, O2, F7, F8, T7, T8, P7, P8, Fz, FCz, Cz, Pz, FC1, FC2, CP1, CP2, FC5, FC, CP5, CP6, FT9, TP9, FT10, and TP10). The factors “group” (2 levels), “channel” (32 levels), and “frequency” (3 levels: alpha, beta, and gamma) were used in the frequency-domain analysis. Degrees of freedom were corrected according to the Greenhouse–Geisser correction when a violation of sphericity was detected. The level of significance was set at *p* < 0.05. Whenever a significant “group”×“channel”×“frequency” interaction was found, three “group”×“channel” interactions were assessed for each frequency and, in case of significance, 32 *post hoc* comparisons between the groups. Their *p*-values were submitted to the false discovery rate procedure to control alpha inflation (Benjamini and Hochberg, 1995).

Due to the small size of pAD-MCI and npAD-MCI subsamples, we did not look for any possible difference between them and instead we focused on the channels in which there was a significant [false discovery rate (FDR), adjusted] *post hoc* difference between the aMCI and controls. On these channels, we performed the rm ANOVA (see results) to determine the possible features differentiating pAD-MCI from npAD-MCI.

To evaluate the GMFP features (# of peaks, aDA, and sDA) and beta and gamma ITC as the predictors of conversion to AD, we trained a binary, linear classification model by using support vector machines, dual stochastic gradient descent, and ridge regularization. Then, we calculated accuracy, sensitivity, and specificity of these parameters (see Supplementary Figure 1 and legend for details).

Finally, we applied the Spearman’s rank correlation test to evaluate the possible relationships between beta or gamma ITC and the # of GMFP peaks (discrete variable) and the Pearson’s correlation test to assess the possible correlations between beta and gamma ITC, sDA, and aDA.

Statistical analyses were conducted by using the STATISTICA (TIBCO Software Incorporation, Palo Alto, CA, United States), whereas the classification analysis was performed by homemade software by using the MATLAB (version 2019b; MathWorks Incorporation).

### Cortical Sources Analysis

Current densities for the representing time points of TMS-induced components were estimated by using the standardized low resolution brain electromagnetic tomography [(sLORETA), Pascual-Marqui et al., 2002] in the Curry software (version 6.0.20, Compumedics Neuroscan, Victoria, Australia) for the illustrative purposes. Current density estimations were analyzed for each group separately and the visualized time points were defined as local maximum values of GMFP. The EEG data and the digitized locations of EEG electrodes were combined with a realistic head model [a three-compartment boundary element model and standard conductivity values (0.33 S/m for the brain fluid, 0.0042 S/m for skull, and 0.33 S/m for skin)] for current source analysis (Ferreri et al., 2016).

## Results

### Amnestic Mild Cognitive Impairment vs. Controls

Age, gender distribution, and education were comparable between the groups, whereas, as expected, aMCI showed the lower MMSE scores than controls (Table 1). RMT and MEPs amplitude were similar between the groups (Table 1).

#### Transcranial Magnetic Stimulation-Evoked Electroencephalogram Responses

**FIGURE 1:**
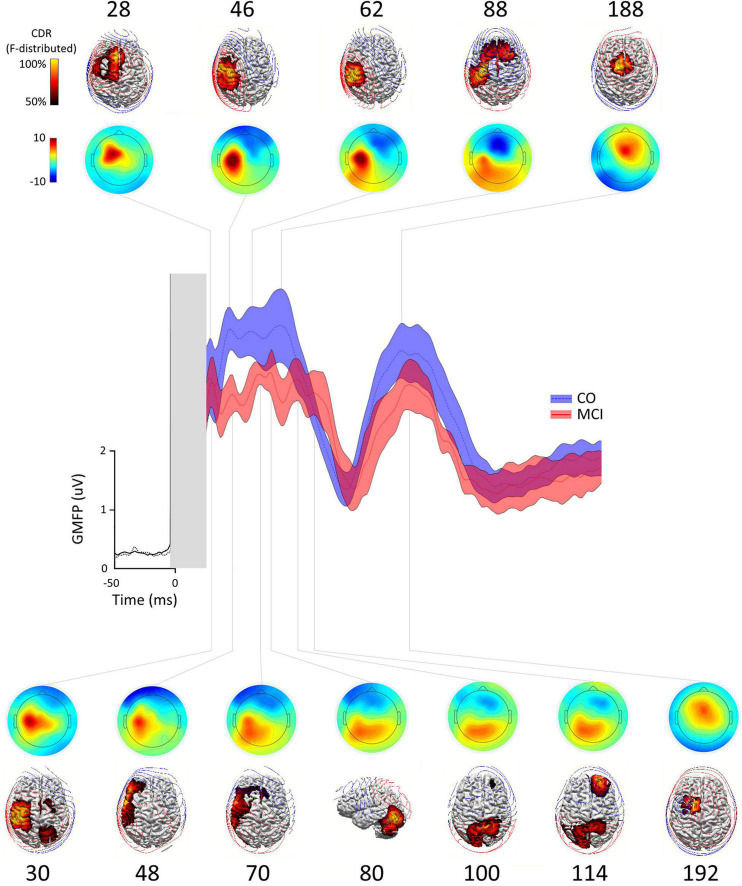
Global mean field power (GMFP), scalp distribution maps, source localizations in control subjects, and the patients with amnestic mild cognitive impairment (aMCI). Scalp distribution maps and source localization of the activity occurring during each peak of the GMFP obtained after the transcranial magnetic stimulation (TMS) of left M1 in healthy subjects (CO, blue trace ± SE) and patients with aMCI (aMCI, red trace ± SE). At each time point, the source localizations were autoscaled and thresholded at 50% to highlight the maximum current sources. In CO, the current maxima (reflecting the maximum neuronal activity) shifted from the stimulated M1 to the ipsilateral premotor/prefrontal cortex border (28 ms), back to the ipsilateral sensory motor cortex (46 ms), becoming more focused on the sensory cortex (62 ms), spreading to the bilateral premotor regions and dorsal prefrontal areas (88 ms), finally focusing on the central midline (188 ms). In MCI, the current maxima at 30 ms poststimulation was still located in the ipsilateral sensory motor cortex expanding to ipsilateral premotor and parietal region and in supplementary motor area (SMA) and contralateral premotor and parietal regions, and then shifted to ipsilateral lateral sensorimotor and lateral premotor and prefrontal regions (48 and 70 ms), to ipsilateral occipito-cerebellar junction, superior parietal lobules bilaterally (100 ms), contralateral prefrontal cortex (114 ms), and finally to ipsilateral premotor region/pre-SMA.

**FIGURE 2:**
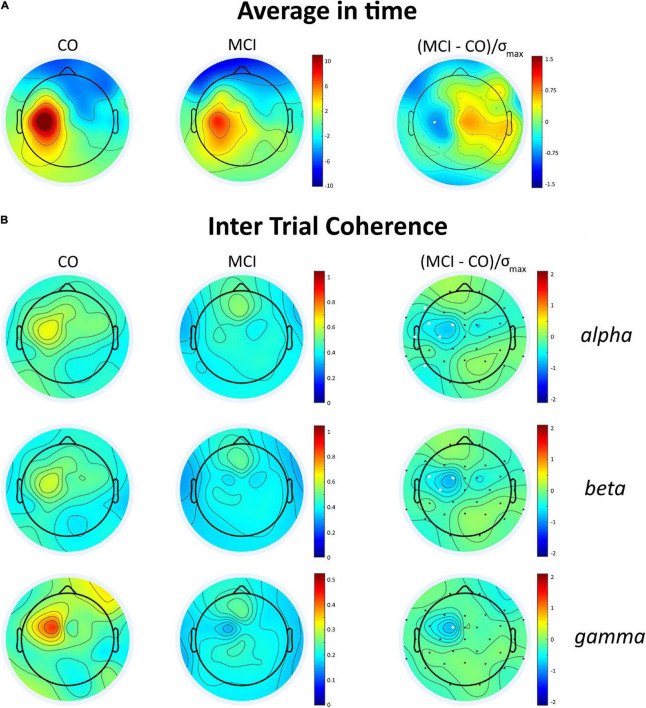
Average integrated TMS-evoked activity and intertrial coherence (ITC) topography in the control subjects and the patients with aMCI. **(A)** Topographic distribution of the average integrated evoked responses in the healthy subjects (CO) and the patients with MCI for the time window 45–55 ms after the TMS over the left M1; the white dot represents the scalp position where the brain excitability is significantly decreased in the patients with aMCI, i.e., C3. **(B)** ITC in the alpha, beta and gamma bands in the time window of 35–145 ms post-TMS in CO and MCI and difference maps (right column). In the difference maps, the white dots represent the scalp position where ITC significantly decreased in the patients with aMCI.

The grand average M1-evoked EEG activity following TMS in the aMCI and controls is shown in Figure 1, along with the source of each GMFP peak. The first (≈30 ms) and last (≈188 ms) GMFP peak were similar between the groups, while the signal differed in amplitude and frequency of the peaks in the time window between the end of the first and the beginning of the last one peak (35–145 ms). In this time window, the analysis revealed a decreased GMFP amplitude at 45–50 ms post-TMS in the aMCI than controls (*p* = 0.045), a latency range corresponding to the N45 TEP (Tremblay et al., 2019). When the map of cortical activity was calculated, a significant “group”×“channel” interaction (*F*_31_,_930_ = 1.859, *p* < 0.01) emerged and *post hoc* analysis indicated lower values in the aMCI than controls in the channel C3 (*p* < 0.001) (Figure 2A). The factor “group” was non-significant (*F*_130_ = 0.386, *p* = 0.54). The frequency analysis showed comparable ERSP values between the aMCI and controls in all the frequency bands, as demonstrated by the non-significant factor “group” (*F*_130_ = 0.08, *p* = 0.78) and the lack of “group”×“frequency”×“channel” (*F*_621_,_860_ = 0.849, *p* = 0.79), “group”×“frequency” (*F*_260_ = 1.757, *p* = 0.18), and “group”×“channel” (*F*_31_,_930_ = 0.707, *p* = 0.88) interactions. Conversely, ITC differed between the groups, as the significant “group”×“frequency”×“channel” interaction (*F*_621_,_860_ = 1.656, *p* < 0.001). The rm ANOVAs conducted for each frequency band revealed topographically specific differences in ITC in the alpha (“group”×“channel” interaction: *F*_31_,_930_ = 1.809, *p* = 0.005), beta (*F*_31_,_930_ = 1.616, *p* = 0.02) and gamma (*F*_31_,_930_ = 1.742, *p* < 0.01) bands. *Post hoc* analyses indicated lower alpha ITC in the aMCI than controls in C3, FC5, F7, T7, P7 (*p* = 0.03), and FC1 (*p* = 0.04); lower beta ITC in C3, FC1, and FC5 (*p* = 0.048); and lower gamma ITC in FC1 (*p* = 0.003) (Figure 2B).

### Prodromal to AD-MCI vs. Non-prodromal to AD-MCI

At the end of the follow-up period, 7 out of 13 subjects with aMCI satisfied the clinical criteria for AD. The median observation time in the patients who remained relatively stable was 69.5 months (see Table 1 for details). At baseline, age, gender distribution, education, MMSE scores, RMT, and MEP amplitude were similar between pAD-MCI and npAD-MCI (Table 1).

#### Transcranial Magnetic Stimulation-Evoked Electroencephalogram Responses

**FIGURE 3:**
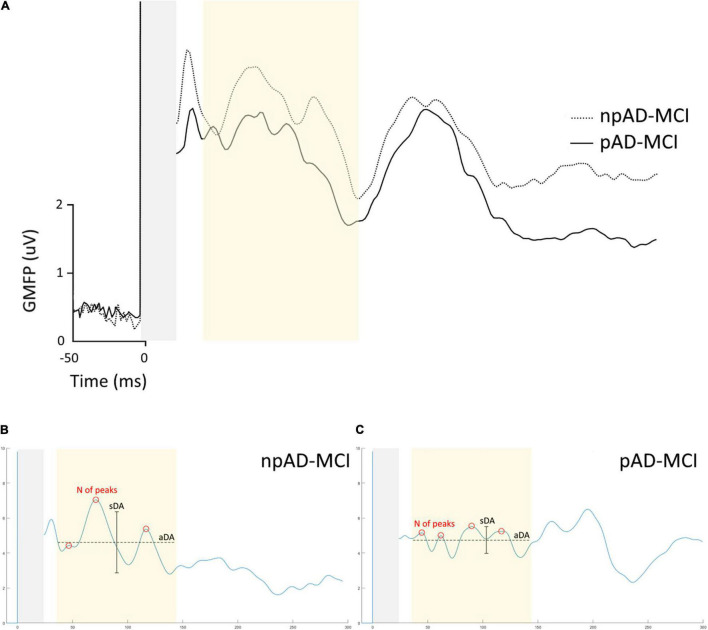
GMFP of pAD-MCI and npAD-MCI groups. **(A)** Average GMFP waveform in the npAD-MCI and pAD-MCI subgroups. **(B,C)** The parameters under evaluation, i.e., the number of peaks, the aDA and the sDA are indicated in the time window of interest in the two paradigmatic subjects, one from each subgroup, for visual purposes.

**FIGURE 4:**
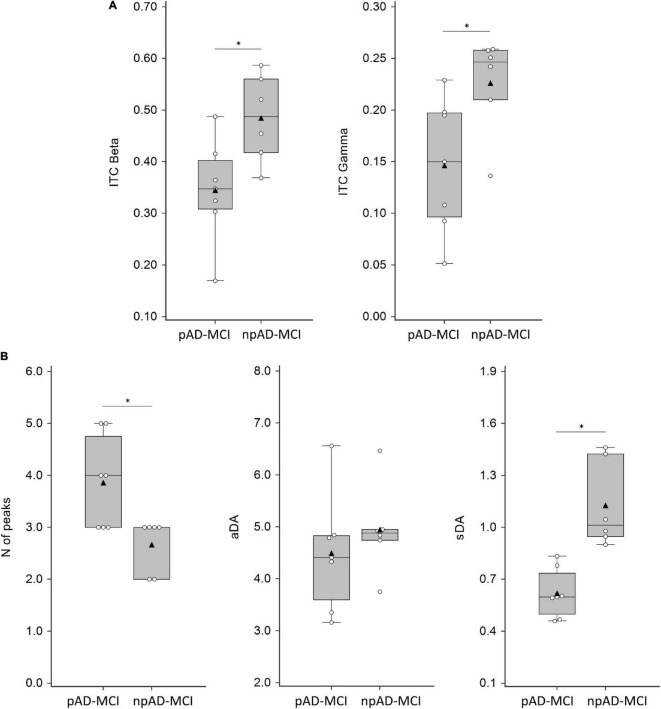
ITC, numbers of peaks, average dipolar activity (aDA), and stability of the dipolar activity (sDA) parameters in MCI groups. **(A)** ITC values in the beta (average of C3, FC1, and FC5 channels) and gamma (FC1 channel) frequency band in pAD-MCI and npAD-MCI. **(B)** Numbers of peaks, aDA, and sDA in pAD-MCI and npAD-MCI. White dots indicate the individual data, while black triangles denote the mean values. Horizontal lines indicate the median value (50th percentile). The boxes contain the 25th to 75th percentiles of dataset. Asterisks indicate significant differences between the groups.

The GMFP in the two subgroups of the patients with aMCI is shown in Figure 3A. We found that some features differentiating the aMCI from controls also diverged between npAD-MCI and pAD-MCI. In particular, pAD-MCI demonstrated lower ITC values than npAD-MCI both in beta (“group”: *F*_111_ = 7.354, *p* = 0.02) and gamma (the unpaired *t*-test: *p* = 0.03) bands (Figure 4A). Conversely, the cortical excitability at 45–50 ms post-TMS (the unpaired *t*-test: *p* = 0.47) and the alpha ITC (“group”: *F*_111_ = 3.084, *p* = 0.11; “group”×“channel”: *F*_555_ = 1.059, *p* = 0.39) were similar between the groups. Moreover, while the GMFP amplitude (*p* > 0.05) and aDA were similar between the subgroups (*p* = 0.44), pAD-MCI demonstrated more GMFP peaks (*p* = 0.048) and lower sDA than npAD-MCI (*p* < 0.001; Figure 4B). Of note, the number of GMFP peaks and sDA in the time windows 25–35 ms (GMFP peaks: *p* = 0.29; sDA *p* = 0.84) and 145–300 ms (GMFP peaks: *p* = 0.36; sDA *p* = 0.98) were similar between pAD-MCI and npAD-MCI. Figures 3B,C show the GMFP metrics in two paradigmatic subjects.

The correlation analysis demonstrated that gamma ITC was positively correlated with sDA (*r* = 0.55, *p* = 0.05) and negatively correlated to the number of GMFP peaks (*r* = 0.57, *p* = 0.04), i.e., the lower the ITC, the higher the number of peaks. Similar correlations, though not statistically significant, were present for beta ITC (beta ITC vs. number of GMFP peaks: *r* = 0.51, *p* = 0.07; beta ITC vs. sDA: *r* = 0.50, *p* = 0.08).

The parameters able to differentiate npAD-MCI and pAD-MCI and, thus, potentially useful to predict the conversion from aMCI to AD according to our classification model are shown in Supplementary Figure 1 and legend.

## Discussion

There are several notable findings in this study. Motor cortex excitability was reduced in the aMCI patients compared to controls, as revealed by the N45 TEP difference. Also, the frequency analysis showed that alpha, beta, and gamma ITC were impaired in the patients, while ERSP in these bands did not change. Notably, while the reduced M1 excitability and alpha ITC were similar in npAD-MCI and pAD-MCI, the impairment in beta and gamma ITC in M1 was significantly stronger in the pAD-MCI subgroup. Finally, sDA, a GMFP parameter reflecting time-specific alterations in TMS-induced activity, discriminated npAD-MCI from MCI who will convert to AD.

Motor cortex hyperexcitability is a well-defined neurophysiological feature of AD (Ferreri et al., 2003, 2011b; Niskanen et al., 2011), which has been recently observed in the whole sensorimotor system (Ferreri et al., 2016). However, this study shows that this feature is not present in the patients with aMCI, even in the pAD-MCI subgroup (Lahr et al., 2016). In aMCI, we observed a cortical hypoexcitability that was time varying, relatively prolonged, and localized onto the stimulated M1. M1 hyperexcitability in AD may reflect both an impairment of cholinergic activity and an imbalance between non-N-methyl-D-aspartate (NMDA) and NMDA neurotransmission in favor to the former. The counterintuitive hypoexcitability in aMCI may reflect increased cholinergic activity (DeKosky et al., 2002) and an elevation of glutamatergic presynaptic bouton density (Bell et al., 2007). In line with this hypothesis, both the cholinergic and glutamatergic systems display a transient upregulation during the MCI stage followed by a successive downregulation with the progression to mild and severe stages of AD. These alterations have been described in frontal cortex, consistently with our localization maps (DeKosky et al., 2002).

Beyond the cortical excitability changes, our analyses in the frequency domain showed a selective ITC disruption and a clear ERSP preservation in the stimulated cortex of the whole aMCI group. This result is particularly intriguing: indeed, ERSP measures the modulation of amplitude induced by a specific event, whereas ITC is an event-phase indicator function strictly related to the concept of signal synchronization in the brain (Delorme and Makeig, 2004). Thus, the result of impaired ITC reflects a rhythm-specific synchronization deficit in the aMCI group. M1 excitability is related to the modulation of oscillatory neural activities in the specific frequency bands, i.e., alpha (Sauseng et al., 2009; Ferreri et al., 2014), beta (Ferreri et al., 2012, 2017b), and gamma (Giustiniani et al., 2019). The analysis showed that the hypoexcitability and decreased alpha ITC were characteristics of aMCI, irrespective of conversion to AD. This lack of specificity can be related to the decrease of alpha activity with age, with a trend similar to brain volume, and robust correlation with aging and cognitive performance (Klimesch, 2012). Moreover, alpha changes do not separate the different etiologies that cause cognitive disruption, with similar changes in MCI due to AD, Parkinson’s disease, and Lewy body dementia (Babiloni et al., 2019). In contrast, reductions in beta and gamma ITC in the stimulated M1 differed between the groups, being more pronounced in pAD-MCI. Hence, beta and gamma M1 ITC reflect specific abnormalities underlying AD pathophysiology. Beta and gamma are the main oscillatory intracortical activities in M1 cortex and they are modulated during the motor control and learning (Guerra et al., 2018a; Bologna et al., 2019). Both the beta and gamma bands are involved in early AD stages and in the prodromal-to-AD condition (Rossini et al., 2020).

Beta oscillations are observed in somatosensory, premotor, supplementary and primary motor cortices. The source of these oscillations remains unclear, but it is clear that they are dependent on intact thalamocortical circuitry (Pfurtscheller et al., 1996). They reflect an idling state of the resting sensorimotor network or, according to a more recent hypothesis, they signal the current motor set at the expense of new movements, being also related to the disengagement of task-irrelevant cortical areas (Engel and Fries, 2010). Finally, beta oscillatory activity is thought to primarily drive interareal connectivity and voluntary activated descending pathways including the corticospinal tract through feed-forward feedback within the cortico-basal ganglia-thalamo-cortical loop (Mantini et al., 2007; Guerra et al., 2021). Gamma oscillations reflect the intracortical neural synchronization processes (Buzsáki and Schomburg, 2015) and have a role in modulating local synaptic plasticity (Guerra et al., 2018b, 2019, 2020; Nowak et al., 2018). Outside the motor system, gamma activity has been related to general cognitive performance, implicit learning, and cognitive component of motor schemes consolidation (Sederberg et al., 2007; Benussi et al., 2021). In M1, it has been involved in planning, synchronization and execution of movements, and in motor learning mechanisms (Bologna et al., 2019; Giustiniani et al., 2019). In addition to its local role in the motor cortices, an emerging function of gamma oscillation is the selective and flexible coupling of neighboring or distant cortical regions including the cortico-basal ganglia-thalamo-cortical loop (Lalo et al., 2008; Fischer et al., 2020).

Thus, the cortico-basal ganglia-thalamic neural circuits are critical modulators of both the motor beta and gamma oscillations. In this context, these oscillations may, thus, represent signals relayed upstream from the basal ganglia to motor cortices and constitute part of a network involved not only in the local actual motor control, but also in providing a long-range spatial and temporal encoding of planned movements. They may synchronize the several involved brain areas and contributing to the conscious awareness of having performed an intended movement (Mantini et al., 2007). In relation to AD, it is notable that motor beta and gamma oscillations are strongly related to the activity of cholinergic interneurons. Ascending cholinergic projections are, indeed, involved in modulation of neuronal activity by rhythms mainly in the beta and gamma ranges in both the short- and long-range connectivity. In mice, the intrinsic physiological striatal acetylcholine (ACh) release modulates beta activity in all the M1 layers and gamma activity in a layer-dependent manner (Kondabolu et al., 2016), with a critical role of striatal muscarinic receptors. There are both *in vivo* and *in vitro* results indicating that ACh plays an important role in modulating the synchronized firing of different neuronal assemblies by a complex activation of both the glutamatergic and gamma-aminobutyric acid GABAergic interneurons (Roopun et al., 2006).

A very important finding of this study concerns the changes observed in the GMFP waveform shape (number and stability of peaks) in a specific time window in pAD-MCI. Interestingly, this time window matched the one in which we found a disrupted TMS-EEG signal transmission in our previous study on patients with AD, possibly subtending altered connectivity of specific networks engaged after the M1 stimulation (Ferreri et al., 2016). The study of the cortical electrical waveforms may offer original information regarding the pathophysiology underlying brain diseases, and novel waveform metrics, providing additional insights into several neurophysiological mechanisms, could be of great general interest (Cole and Voytek, 2017). Under this assumption, we investigated if waveform shape detected with TMS-triggered EEG responses and globally represented by the GMFP might be an electrophysiological biomarker of the MCI prodromal-to-AD condition. We found that the stability of the dipolar activation (sDA) in the GMFP in a specific time window had high sensitivity, specificity, and accuracy in identifying, in our dataset, those subjects with aMCI who converted to AD in the following 6 years. Therefore, it could be considered worthy of further investigation in a larger cohort to evaluate its role in predicting the conversion from aMCI to AD. This parameter positively correlated with gamma frequency ITC, i.e., the lower the coherence, the lower the stability of the cortical dipolar activation. Considering these results, we speculate that in our patients with pAD-MCI, the reduced cortico-basal ganglia-thalamo-cortical loop synchronization in high frequencies may constrain neurons in a chaotic pattern inhibiting the disengagement of task-irrelevant cortical areas and finally leading to a disruption of neural communication that arise in a disrupted cortical electrical waveforms (Jackson et al., 2019). This would be also in line with the correlation between the gamma frequency ITC and the numbers of GMFP peaks, i.e., the lower the coherence, the higher the number of peaks.

Recent studies showed that peripheral evoked potentials (PEPs) can contaminate TEPs (Tremblay et al., 2019). We believe that this was unlikely to occur in this study because we use the current state-of-art procedures to minimize this issue (Rocchi et al., 2020). Moreover, PEPs are known to affect late, but not early, TEPs (Rocchi et al., 2020) and their frequency components fall in the low range of the spectrum, while our results demonstrated altered N45 and beta-to-gamma ITC in the patients.

This study has some limitations. First, since the number of participants was low, the results should be considered as preliminary. Particularly, the small sample size precludes to consider the estimates of sensitivity, specificity, and accuracy of our TMS-EEG parameters as reliable. The estimation of sDA and gamma ITC accuracy should be considered as a suggestion of their potential role as predictors of AD conversion in a proof of principle study and need to be confirmed in an independent and larger validation sample. Moreover, even though the classification of aMCI patients in prodromal and non-prodromal to AD was based on repeated neuropsychological evaluations along a prolonged follow-up period (Rossini et al., 2019, 2020), biological markers were not available to support a precise disease etiology. Future studies are needed to assess whether these neurophysiological abnormalities are specific to the subjects with aMCI who have biological markers of AD pathology. Finally, although the median clinical-neuropsychological follow-up time for npAD-MCI patients was 6 years, we cannot fully exclude a later cognitive/functional worsening.

## Conclusion

Motor symptoms are considered late events in the natural history of AD and their early occurrence makes the diagnosis less likely. However, there is a growing body of neuropathological evidence that M1 is already involved in the early AD stages, despite the lack of clinically evident motor deficits and the reasons for this discrepancy are still matter of debate (Ferreri et al., 2003, 2011b; Di Lorenzo et al., 2020). Within this theoretical framework, we aimed at investigating the TMS-EEG hallmarks of sensorimotor cortex functionality in aMCI, assuming some represent the subtending plastic rearrangement induced by the neurodegeneration and could, thus, predict the conversion to AD (Ferreri and Rossini, 2013). We demonstrated selective- and region-specific alterations of M1 functionality, which may reflect underlying neurodegenerative processes and possibly predict the evolution from aMCI to AD.

## Data Availability Statement

Preprocessed and anonymized data will be made available upon reasonable request to the corresponding authors.

## Ethics Statement

The studies involving human participants were reviewed and approved by Campus Bio-Medico University of Rome. The patients/participants provided their written informed consent to participate in this study.

## Author Contributions

FF and PR conceived the study. FF, DP, and PR designed the experiments with input from all the other authors. FF, AG, FV, and FM performed TMS-EEG experiments. AG and LV performed TMS-EEG data analysis and statistics with assistance from FF, IS, and DP generated related figures. LV set the linear classification model. SM and MK performed the sLORETA analysis and generated related figures. FF, AG, and PR screened and clinically followed the patients with MCI. FF, AG, LV, and DP wrote the manuscript with editorial input from all the other authors. PP supervised the statistical analysis. PR and MC supervised the study. All authors contributed to the article and approved the submitted version.

## Conflict of Interest

The authors declare that the research was conducted in the absence of any commercial or financial relationships that could be construed as a potential conflict of interest.

## Publisher’s Note

All claims expressed in this article are solely those of the authors and do not necessarily represent those of their affiliated organizations, or those of the publisher, the editors and the reviewers. Any product that may be evaluated in this article, or claim that may be made by its manufacturer, is not guaranteed or endorsed by the publisher.

## Supplementary Material

Supplementary Figure 1 Classifier scatter plot results and robustness of the classification results. **(A)** Scatter plot results show the relationship between the parameters able to distinguish npAD-MCI and pAD-MCI in predicting conversion to Alzheimer’s disease at the individual level. The graphs highlight the performance of such features. The binary, linear classification model we used was validated through a cross-validation approach based on the leave-one-out method. The best performing parameters and the best combination of parameters in distinguishing between pAD-MCI and npAD-MCI are highlighted in red. Accuracy, sensitivity, and specificity of the various parameters were calculated using as cutoffs those identified according to the maximum Kolmogorov–Smirnov test. In order to test accuracy, the Clinical and Laboratory Standard Institute recommends to have ≥50 cases to assess sensitivity and 50 controls to assess specificity. This is a proof-of-principle study and does not satisfy such sample size. However, in order to provide information about the precision of our estimates, 95% confidence interval (CIs) were calculated. Specifically, we applied Wilson’s method, since it allows to calculate CIs even in the case of 100% sensitivity and specificity, differently from the binomial exact method. The parameters showing the highest sensitivity, specificity, and accuracy were sDA and gamma ITC. In particular, sDA (cutoff = 0.86) demonstrated a sensitivity of 100% (95% CI: 72–100%), specificity of 100% (95% CI: 69–100%), and accuracy of 100% (95% CI: 77–100%), while gamma ITC (cutoff = 0.20) showed a sensitivity of 86% (95% CI: 49–97%), specificity of 83% (95% CI: 44–97%), and accuracy of 85% (95% CI: 58–96%). **(B)** To investigate the robustness of our classification model, we performed an evaluation based on the progressive random drop of trials, repeated 100 times for each drop percentage and the reproduction of all the steps required to build and test the classifiers from the extraction of the parameters which performed best. This analysis showed that the performance of the classification process degrades slowly and it suggests the robustness of the results.
