# Supplementary figures and images for: TMS-EEG Biomarkers of Amnestic Mild Cognitive Impairment Due to Alzheimer’s Disease: A Proof-of-Concept Six Years Prospective Study
Source: Front Aging Neurosci. 2021 Nov 22;13:737281. doi: 10.3389/fnagi.2021.737281 (PMC8645846; doi:10.3389/fnagi.2021.737281)

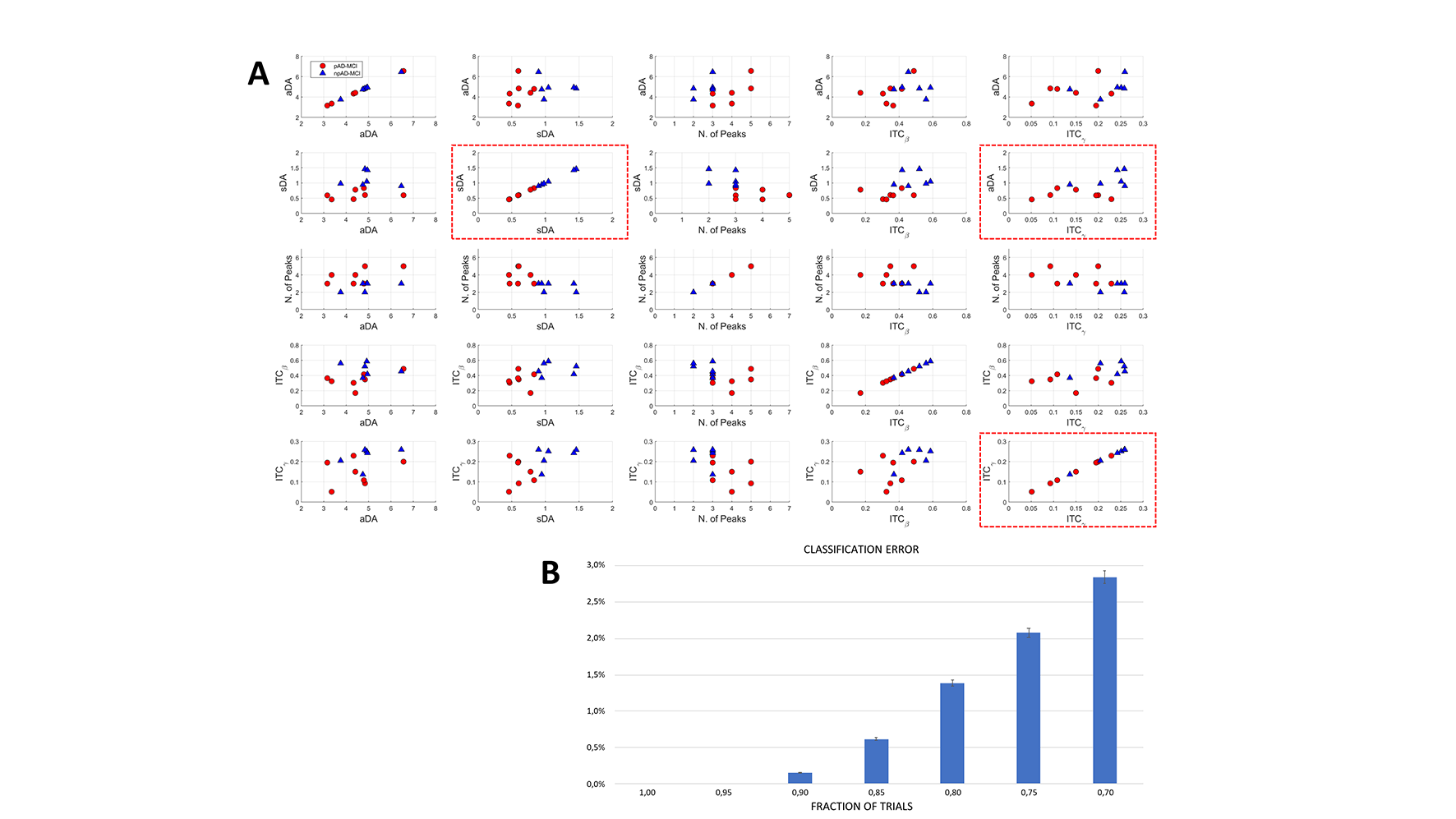

Supplement: Supplementary Figure 1 — Classifier scatter plot results and robustness of the classification results. (A) Scatter plot results show the relationship between the parameters able to distinguish npAD-MCI and pAD-MCI in predicting conversion to Alzheimer’s disease at the individual level. The graphs highlight the performance of such features. The binary, linear classification model we used was validated through a cross-validation approach based on the leave-one-out method. The best performing parameters and the best combination of parameters in distinguishing between pAD-MCI and npAD-MCI are highlighted in red. Accuracy, sensitivity, and specificity of the various parameters were calculated using as cutoffs those identified according to the maximum Kolmogorov–Smirnov test. In order to test accuracy, the Clinical and Laboratory Standard Institute recommends to have ≥50 cases to assess sensitivity and 50 controls to assess specificity. This is a proof-of-principle study and does not satisfy such sample size. However, in order to provide information about the precision of our estimates, 95% confidence interval (CIs) were calculated. Specifically, we applied Wilson’s method, since it allows to calculate CIs even in the case of 100% sensitivity and specificity, differently from the binomial exact method. The parameters showing the highest sensitivity, specificity, and accuracy were sDA and gamma ITC. In particular, sDA (cutoff = 0.86) demonstrated a sensitivity of 100% (95% CI: 72–100%), specificity of 100% (95% CI: 69–100%), and accuracy of 100% (95% CI: 77–100%), while gamma ITC (cutoff = 0.20) showed a sensitivity of 86% (95% CI: 49–97%), specificity of 83% (95% CI: 44–97%), and accuracy of 85% (95% CI: 58–96%). (B) To investigate the robustness of our classification model, we performed an evaluation based on the progressive random drop of trials, repeated 100 times for each drop percentage and the reproduction of all the steps required to build and test the classifiers from the extraction of th [file Image_1.tif]
